# The correlation between sperm percentage with a small acrosome and unexplained in vitro fertilization failure

**DOI:** 10.1186/s12884-023-06205-0

**Published:** 2024-01-11

**Authors:** Chuyan Li, Ya Ni, Lingnv Yao, Jiajie Fang, Nan Jiang, Jing Chen, Wenqin Lin, Hanchen Ni, Haiyan Zheng

**Affiliations:** 1 Center for Reproductive Medicine, The First Affiliated Hospital of Zhejiang University School of Medicine, Hangzhou, Zhejiang Province 310013 China; 2 Urinary Surgery, The First Affiliated Hospital of Zhejiang University School of Medicine, Hangzhou, Zhejiang Province 310013 China; 3 Reproductive Physiology Laboratory, Hangzhou Medical College/Zhejiang Academy of Medical Sciences, Hangzhou, Zhejiang Province 310013 China

**Keywords:** Unexplained in vitro fertilization failure, Acrosome, Acrosin activity, Acrosome reaction

## Abstract

**Purpose:**

Since the unexplained in vitro fertilization failure occurs frequently, it is of great importance and clinical value to identify potential underlying predictors. This study aimed to explore whether the percentage of sperm with a small acrosome was correlated with unexplained in vitro fertilization failure.

**Methods:**

A new acrosomal function evaluation index (the percentage of sperm with a small acrosome) was introduced into the analysis of sperm morphology. The association between the index and acrosome function by acrosin activity detection test and acrosome reaction test was investigated. In addition, the correlation with unexplained in vitro fertilization failure was further explored. Finally, the ROC curve was used to analyze the diagnostic efficacy on the failure of in vitro fertilization and the cutoff value was calculated.

**Results:**

As the increasing of the percentage of sperm with a small acrosome, the value of acrosin activity, acrosome reaction rate, and in vitro fertilization rate were reduced, with a statistically significant difference (P < 0.05). The index in the low fertilization rate group was significantly higher than that in the normal fertilization rate group (P < 0.05). Finally, the results of ROC curve found that when the index was 43.5%, the sensitivity and specificity were 74.2% and 95.3%, respectively.

**Conclusion:**

The percentage of sperm with a small acrosome was positively correlated with unexplained in vitro fertilization failure, which could be potentially used as a prognostic index for the failure of in vitro fertilization.

**Trial registration:**

[Ethics review acceptance No IIT20210339B]

## Background

Approximately 10–15% of couples of childbearing age worldwide suffer from infertility. Although the majority of infertile couples are able to conceive successfully through in vitro fertilization (IVF), failures (less than 30% conception rate) or complete failures do occur from time to time [1–3]. Rescue intracytoplasmic sperm injection (R-ICSI) is an assisted reproductive technique in which a single sperm is injected into the cytoplasm of the oocyte by microscopic manipulation, and is the only remedy after in vitro fertilization has failed [4]. Recent studies have shown that early remedial intracytoplasmic single sperm microinjection (ICSI) has a fertilization rate of 24–48% in oocytes that have failed to fertilize. However, because ICSI allows oocytes to be fertilized with sperm cells that may not be competent, there is growing concern about male inheritance patterns [5]. Failure of fertilization not only increases the financial burden, but also causes psychological stress to the patient. Therefore, identifying potential predictors of in vitro fertilization failure is important for clinical practice.

The acrosome is an important structure of the sperm head, accounting for 40% ~ 70% of its volume, and is rich in polysaccharides and various hydrolases [6]. The acrosome function evaluation of sperm is a core feature of the evaluation of sperm fertilization ability. After the encounter between the capacitated sperm and ovum, contact between the sperm and ovum triggers an acrosome reaction [7, 8]. Currently, there are several methods for assessing sperm acrosome function. First is the zona-free hamster egg sperm penetration assay (SPA). Although this method has high accuracy, it has the disadvantages of time-consuming and expensive, making it unsuitable for routine evaluation. Instead, it is often used to verify the effectiveness of new detection methods in evaluating sperm fertilization function [9, 10]. In addition, the acrosome reaction (AR) is mostly used in scientific research due to time-consuming and laborious method [11–13]. The hyaluronan binding assay (HBA) has not been used as a routine clinical evaluation method [14]. In contrast, the zona pellucida glycoprotein-3 (ZP3) co-test is a new method that needs to be further explored before clinical application [15]. Last one is the acrosin activity detection test, which is most commonly used to evaluate sperm acrosome function in clinical practice [16, 17]. However, this test method is only applicable to fresh semen, and the operation is cumbersome and time-consuming. Furthermore, this method could only be used as a reference index to evaluate sperm acrosome function, but not for the selection of pregnancy aid method. Therefore, a simple and accurate index to evaluate the acrosome function of sperm is urgently needed.

Sperm morphology analysis is a routine test in many fertility centers, but the proportion of small acrosomal spermatozoa (acrosomes make up less than 40% of the sperm head) is not measured separately. In this study, the percentage of sperm with a small acrosome was introduced into the morphological analysis, and the correlation between this index and acrosome function (such as acrosin activity detection and acrosome reaction) was analyzed. In addition, the correlation between the percentage of sperm with a small acrosome and unexplained failure of in vitro fertilization was evaluated.

## Materials and methods

### Reagents and medium

Chlortetracycline (CTC), L-cysteine, BSA (Fraction V), and Hoechst 33,258 were obtained from Sigma (St. Louis, MO, USA). To prepare CTC, 10 ml of chilled buffer of Tris (20 mM) and NaCl (130 mM) (TN) was added to 8.8 mg L-cysteine followed by 2.6 mg CTC. The CTC solution was vortexed for 30 s, adjusted to pH 7.8 with 1 M NaOH, and placed on ice. This solution was freshly prepared prior to use. Sperm morphology staining reagent was purchased from Zhuhai Beso Biotechnology Co. Ltd. (cat. no. C201201). The sperm acrosomal enzyme activity assay kit was purchased from Shenzhen Huakang Biomedical Engineering Co., Ltd. (cat. no. 20,210,801).

Human tube fluid (HTF) was used in this study; 100 ml HTF medium consisted of 584.6 mg NaCl, 37.0 mg KCl, 26.5 mg CaCl2 (2H2O), 24.9 mg MgSO4 (7H2O), 210.5 mg NaHCO3, 110.2 mg glucose, 10 mM sodium lactate, 2.97 mg sodium pyruvate, 15.92 mg KH2PO4, 400 mg bovine serum albumin (fraction V) and 6 mg penicillin. This medium had a final osmolality of 305–310 mOsm/kg and pH 7.5 at room temperature (25 °C).

### Research subjects

A total of 512 couples (female age 33±5.4 years and male age 34±4.8 years) underwent IVF assisted conception treatment at our Centre for Reproductive Medicine between October 2016 and August 2021, and a total of 512 semen samples were collected from male patients. Inclusion criteria: (i) couples undergoing IVF treatment for the first time; (ii) infertility due to tubal factors in women and normal routine semen examination in men. Exclusion criteria: (i) couples in which the presence of anti-sperm antibodies was observed by direct immunobead binding test evaluation; (ii) the presence of reproductive disorders such as polycystic ovary syndrome and endometriosis. The study was approved by the Human Ethics Committee of the First Affiliated Hospital of Zhejiang University (Ethics review acceptance No IIT20210339B), each patient signed an informed consent form to participant in the study. The questionnaire was completed excluding other factors. These included age (women were included up to the age of 39 and men up to the age of 55), working conditions (occupations affecting fertility, such as those involving chemicals and high levels of radiation, were excluded), dietary habits, smoking and health status.

### Methods

#### Determination of the percentage of sperm with a small acrosome

Semen samples were collected through masturbation. The semen sample was divided into two parts, one for subsequent experimental testing. The other part follows the procedure described by Evenson [18], storing the raw semen in Eppendorf bayonet tubes in a -80 °C ultra-cold refrigerator for subsequent in vitro fertilization. To avoid any possible confounding effects during fixation and staining, the shape of the acrosomes of fresh live spermatozoa was analyzed using a sperm morphometric analysis (SMA) algorithm, where spermatozoa with acrosomal coverage of < 40% of the volume of the normal head anterior were counted as acrosomal microspermatozoa [19].

Preparation: After routine semen analysis, two smears were taken for each specimen according to the pulling technique provided in the WHO Manual for Human Semen Examination and Handling Laboratories, 5th Edition, and then air-dried.

Staining: Reagent preparation and dyeing were carried out according to the methods provided in the WHO Laboratory Manual for Human Semen Examination and Treatment, 5th Edition. The prepared smears were fixed with 95% ethanol for 15 min, and then stained. The dyeing steps were as follows: 80% ethanol for 30 s; 50% ethanol for 30s; distilled water for 30s; hematoxylin stain for 4 min; distilled water for 30s; immersed in acid ethanol 4 ~ 8 times, each lasting 1 s; cold water for 5 min; 50% ethanol for 30s; 80% ethanol for 30s; 95% ethanol for 30s; G6 orange for 1 min; 95% ethanol for 30s three times; EA-50 stain for 1 min; 95% ethanol for 30s two times; 100% ethanol for 15s two times; finally, air-dried.

Film reading: observation under 100X microscope with oil, analysis of at least 500 spermatozoa, and calculation of the percentage of sperm with a small acrosome = count of sperm with acrosome < 40% of sperm head /total sperm count.

### Sperm morphological staining

In a sperm with a normal acrosome (N), the acrosome accounted for > 40 of sperm head; in a sperm with a small acrosome (S), the acrosome accounted for < 40% of the sperm head. The percentage of sperm with a small acrosome was quantified in this study: S / total sperm count X 100%.

**Fig. 1:**
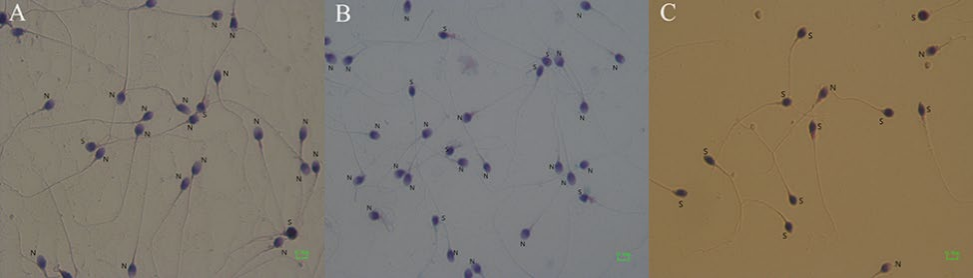
The percentage of sperm with a small acrosome. S stands for small acrosome and N for acrosome. **A**: the percentage < 20% **B**: the percentage 20–40% **C**: the percentage > 40%. Picture magnification is 100x

A total of 512 semen samples from the males of infertile couples were divided into three groups: group A (266 specimens, Fig. 1A), with the percentage of sperm with a small acrosome < 20%; group B (171 specimens Fig. 1B), with the percentage of sperm with a small acrosome, 20–40%; group C (75 specimens Fig. 1C) the percentage of sperm with a small acrosome > 40%.

### Sperm acrosin activity detection

The kit produced by Shenzhen Huakang Biomedical Engineering Co., Ltd., was used for sperm acrosin activity determination. The activity of arginine amidase was calculated by measuring the amount of the colored product, benzoyl-DL-arginate-P-nitroaniline (BAPNA), which was decomposed using Na-BAPNA as the substrate.

After the fresh semen samples were liquefied, the sperm concentration (M×106 /mL) was calculated. Semen volume (V = 7.5 /M) was calculated according to the semen concentration (M×106 /mL). The supernatant was discarded after centrifugation (2000 g ×20 min); 100 µL inhibitor was added to both the determination tubes and control tubes. After mixing, 1000 µL reaction solution was added to both tubes and another 100 µL stop solution was added to the control tubes. After incubation at 24 °C for 1 h, 100 µL stop solution was added to the determination tube and centrifuged both tubes (2000 g × 15 min). Then, 0.5 cm colorimetric dish and 410 nm wavelength colorimetric was used to read the OD value, and the acrosomal enzyme activity of semen samples was calculated. The normal reference values were 48.2 ~ 218.7 µIU/106 spermatozoa.

#### Acrosome reaction

The sperm density was adjusted to 2–3 × 107 cells/ml. Spermatozoa were cultured at 37 °C with 5% CO2 for 5 h. After the spermatozoa were centrifuged (500 g, 5 min), the supernatant was discarded. HTF was suspended and the density was adjusted to 2–3 × 107 cells/ml; 20 µg/ml rhuZP3a was added and incubated (37 °C, 5% CO2) for 30 min. An equal volume of 8% paraformaldehyde was added to each specimen, for a final concentration of 4%, and fixed at 37 °C for 5 min. An equal volume of prepared CTC was added to each group and pre-stained at 37 °C for 15 min. Hoechst 33,258 was added to each specimen to a final concentration of 1 µg/ml in the dark for 2 min to distinguish dead sperm from living sperm. Samples were centrifuged (500 g, 5 min), the supernatant was discarded, and the specimens were suspended in 20 µl HTF and pressed into tablets. A phase contrast microscope and fluorescence microscope were used for cross-observation with a 100X objective lens. A total of 500 spermatozoa were observed in each group and the acrosome reaction rate (AR%) = number of spermatozoa with acrosome reaction/total number of spermatozoa × 100% was calculated.

### Routine IVF

Controlled ovulation induction program: This program lasted for 20–24 days prior to the menstrual cycle. Gonadotropin releasing hormone agonist (GnRHa) was administered, and gonadotropin (Gn) was administered to initiate stimulation after the pituitary gland was stimulated. When the dominant follicles reach the trigger standard, Gn was stopped and HCG was injected at night. After 34–36 h, the eggs was taken by vaginal puncture under B-ultrasound guidance.

IVF and embryo observation: The regimen lasts 20–24 days prior to the menstrual cycle, and controlled ovarian stimulation was achieved using either a GnRH antagonist short protocol or a GnRH-agonist down-regulated long protocol. Gonadotropin-releasing hormone agonist (GnRHa) was injected and gonadotropins (Gn) were injected after stimulation of the pituitary gland to initiate stimulation. When the dominant follicle reaches the trigger criteria (when 1 dominant follicle reaches 20 mm or at least 3 follicles reach 18 mm in diameter), Gn is stopped and HCG is injected at night. After 34–36 h, eggs were retrieved by vaginal puncture under ultrasound guidance. In vitro fertilization and embryo observation: Eggs were obtained and incubated in an incubator for 3–4 h. After washing, semen was added and incubated. Fertilization was observed after a total of 16–18 h of incubation. Normal fertilization is 2PN (2 prokaryotes and dipole), while 1PN (1 prokaryote) or multiple PN (more than 2 prokaryotes) produces abnormal fertilization. Fertilization rate = normal oosperm /total number of mature oocytes. In order to reduce the effect of oocyte maturity on fertilization, the denominator for fertilizations rate used in the study was mature oocytes rather than total oocytes.

### Statistical analysis

**Fig. 2:**
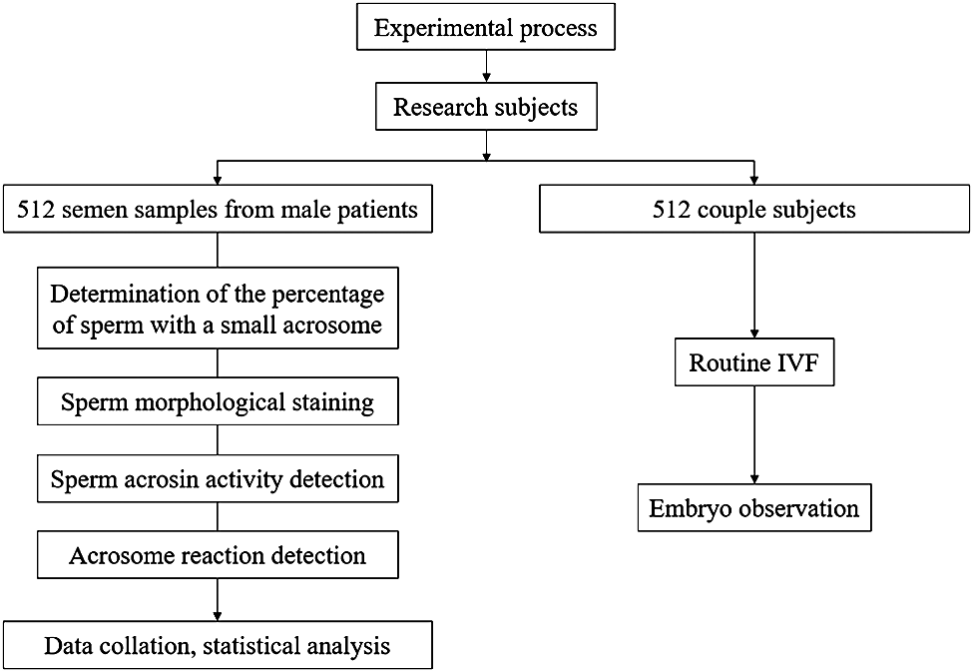
Research flowchart

SPSS 22.0 software was used for statistical analysis. The results were expressed as mean ± SD. A two-tailed Student’s t-test was used for statistical analysis. For three or more groups, data were analyzed using one-way ANOVA and Dunnett’s post hoc test, respectively. The ROC curve was used to analyze the diagnostic efficacy of the index. P < 0.05 was regarded as statistical significance. The specific research process is shown in Fig. 2.

## Results

### The percentage of sperm with a small acrosome and acrosin activity

Acrosin activity was determined after liquefaction. The results showed that with an increase in the percentage of sperm with a small acrosome, the value of acrosin activity was lower, and the difference was statistically significant (P < 0.05) (Table 1).

**Table 1:** Correlation of sperm percentage with acrosin activity

Percentage of sperm with a small acrosome	n	acrosin activity (µIU/ml)	95% (CI)	*P*
< 20%	266	151.7 ± 48.8	145.7-157.6	0.000
20–40%	171	90.8 ± 18.2	88.0-93.53	0.000
> 40%	75	40.9 ± 12.9	37.5–43.5	0.000

### The percentage of sperm with a small acrosome and acrosome reaction

**Fig. 3:**
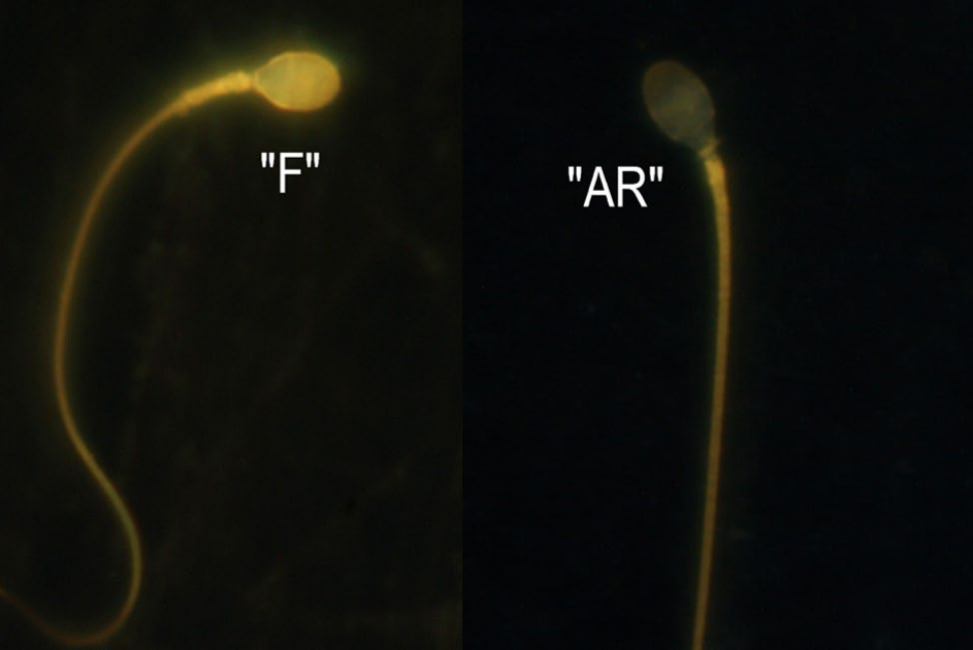
The patterns of chlortetracycline staining in human spermatozoa during capacitation. The ‘F’ pattern was characteristic of non-capacitated, acrosome-intact spermatozoa; and the ‘AR’ pattern was characteristic of acrosome reacted spermatozoa

The spermatozoa were divided into dead sperm (the whole head of sperm was stained blue) and living sperm, which can be divided into three categories: “F” type (non-capacitated sperm, the whole acrosome was stained yellow); “B” type (capacitated but no acrosome reaction sperm, the acrosome was present, but a faint fluorescent zone appeared in the region behind the acrosome); and “AR” type (acrosome reactive sperm, the whole head was pale yellow or non-fluorescent). The tails of all spermatozoa were yellow (Fig. 3). “AR” % was calculated as “AR” type sperm / total sperm count × 100%.

Acrosome reaction was determined after liquefaction of semen samples. The results showed that the percentage of sperm with a small acrosome was negatively associated with the AR%. The difference was statistically significant (p < 0.05; Table 2).

**Table 2:** Correlation of sperm percentage with acrosome reaction

Percentage of sperm with a small acrosome	n	acrosome reaction(%)	95% (CI)	*P*
< 20%	266	49.9 ± 6.6	49.7–50.7	0.000
20–40%	171	30.0 ± 7.4	28.9–31.2	0.000
> 40%	75	18.1 ± 4.8	17.0-19.2	0.000

### In vitro fertilization (IVF) rate

**Fig. 4:**
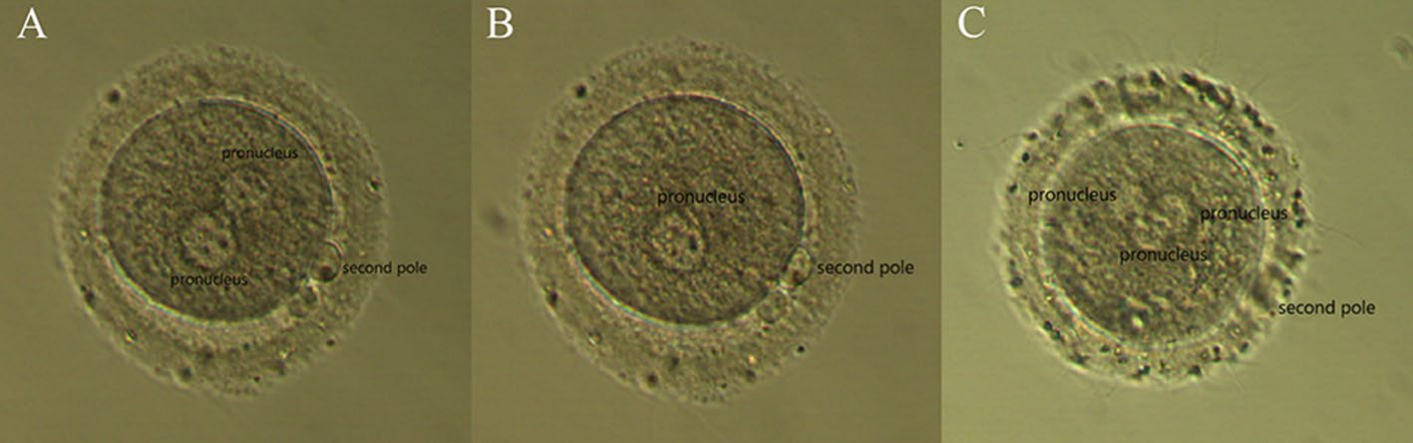
Results of fertilization. **A**: Double pronucleus **B**: Single pronucleus **C**: Multiple pronucleus

Fertilization was observed 16–18 h after coculture. Normal fertilization was 2PN (2 prokaryotes and dipoles, Fig. 4A), while 1PN (one prokaryote, Fig. 4B) or multiple PN (more than two prokaryotes Fig. 4C) showed abnormal fertilization. The semen samples of the male partner from 512 couples were divided into two groups according to the IVF fertilization rate (Normal: fertilization rate > 30%, 433 samples; Low: fertilization rate ≤ 30%, 79 samples). The results showed that the percentage of sperm with a small acrosome in the low fertilization rate group was significantly higher than that in the normal fertilization rate group (P < 0.05) (Table 3).

**Table 3:** Correlation of sperm percentage with unexplained IVF fertilisation failure

Fertility rate	n	percentage of sperm with a small acrosome	95% (CI)	*P*
> 30%	433	23.0 ± 5.3	22.5–23.5	0.000
≤30%	79	47.0 ± 8.3	45.1–48.8	0.000

### The percentage of sperm with a small acrosome and IVF fertilization rate

The results indicated that the in vitro fertilization rate decreased as the increasing of the percentage of sperm with a small acrosome. There was a significant negative correlation between IVF fertilization rates and the percentage of sperm with a small acrosome (P < 0.05) (Table 4).

**Table 4:** Correlation between sperm percentage and IVF fertilization rate

Percentage of sperm with a small acrosome	n	IVF fertilization rate (%)	95% (CI)	*P*
< 20%	266	91.7 ± 11.6	90.4–93.2	0.000
20–40%	171	72.9 ± 13.4	70.9–74.9	0.000
> 40%	75	39.8 ± 7.6	38.1–41.5	0.003

**Fig. 5:**
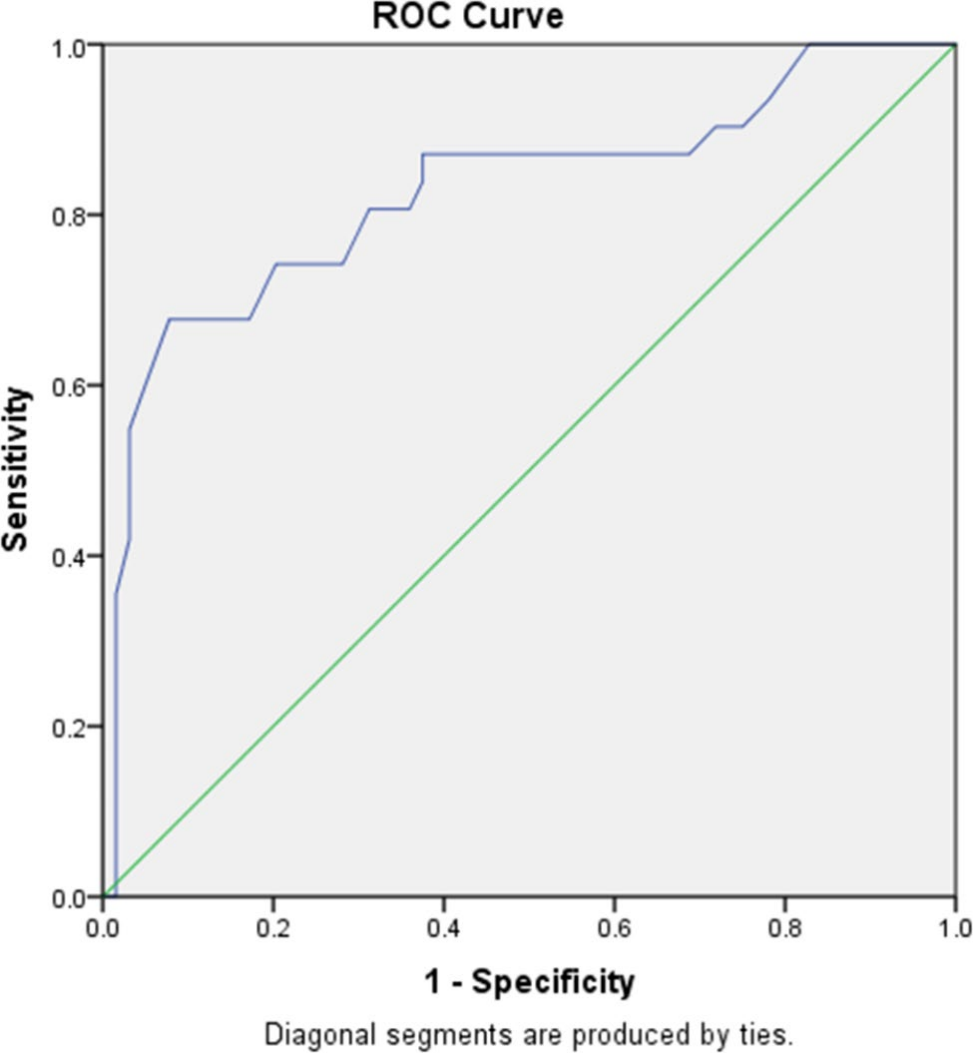
ROC curve of the percentage of sperm with a small acrosome predicting IVF fertilization failure

The area under the curve for predicting fertilization failure was 0.889 (95% CI: 0.813–0.966). The optimal critical value was 43.5%, where the sensitivity and specificity were 74.2% and 95.3%, respectively, as shown in Fig. 5.

## Discussion

This study found that the percentage of sperm with a small acrosome was negative correlated with the value of acrosin activity, acrosome reaction rate, and in vitro fertilization rate. In addition, the percentage of sperm with a small acrosome was positively correlated with unexplained in vitro fertilization failure, which could be potentially used as a prognostic index for the failure of in vitro fertilization.

The acrosome is the cap-like region in the front two-thirds of the head of mature sperm, which contained acrosomal enzymes [20]. Acrosomal enzymes consist of a variety of hydrolases, such as acrosomal protease, hyaluronidase, and acid phosphatase. During fertilization, sperm release these acrosomal enzymes to decompose the radial crown and zona pellucida in the periphery of the ovum, thus helping the sperm to break through this barrier and form a passage into the ovum [21]. Therefore, the evaluation of the acrosome function of sperm is very important for the selection of fertilization methods. The most common method of evaluating sperm acrosome function is the detection of sperm acrosin activity in clinic practice. Although research has suggested that abnormal sperm acrosin activity is a potential cause of infertility [16, 22, 23], this test can only be applicable to fresh semen, and the procedure is cumbersome and time-consuming. Therefore, the sperm acrosomal enzyme measurement is only used to evaluate sperm acrosome function, instead of assisted reproduction method selection.

By measuring acrosin activity and the AR% among different groups of the percentage of sperm with a small acrosome, we found that as the increasing in the percentage of sperm with a small acrosome, the acrosin activity and the AR% was reduced. This negative correlation was statistically significant (P < 0.05). Previous evidence showed that the higher the percentage of sperm with a small acrosome, the lower the value of acrosomal enzymes, and the lower the acrosome reaction rate. Thus, the percentage of sperm with a small acrosome can be used to predict acrosome function.

Currently, R-ICSI is the only remedy after failure of in vitro fertilization. Different methods of assisted reproduction are chosen according to the routine sperm parameters. IVF-ET can be chosen for those with normal or slightly low routine sperm parameters, and ICSI can be chosen for those with severe abnormal routine sperm parameters. However, even with normal sperm from routine examinations for IVF, fertilization failure often occurs, a large part of which are considered to be unexplained fertilization failure [24, 25]. In recent years, with the development on the mechanisms of male infertility, various causes of fertilization failure were found. Therefore, it is necessary to explore related predictive indicators of in vitro fertilization, which have important scientific significance and clinical value. The study found that the percentage of sperm with a small acrosome in the low fertilization rate group was significantly higher than that in the normal fertilization rate group, which indicated that the percentage of sperm with a small acrosome could be a potential novel predictor for unexplained fertilization failure.

Receiver operating characteristic (ROC) curve analysis, an important tool for evaluating the merits and disadvantages of diagnostic experiments. An area below 0.5 indicates that the experiment has no diagnostic value; between 0.5 and 0.7 is low accuracy; 0.7–0.9 is moderate accuracy; and above 0.9 is high accuracy [26]. In this study, an ROC curve was used to analyze the diagnostic efficacy of the percentage of sperm with a small acrosome on in vitro fertilization failure, and to find the cut-off value, and the prediction index of in vitro fertilization failure. The results of the study showed that the area under the ROC curve was 0.889, which was close to a high diagnostic value for fertilization failure. The ROC curve of the percentage of sperm with a small acrosome to in vitro fertilization failure showed that when the percentage of sperm with a small acrosome was 43.5%, the sensitivity and specificity were 74.2% and 95.3%, respectively, indicating that the percentage of sperm with a small acrosome had a high diagnostic value. Therefore, the percentage of sperm with a small acrosome could be used as a new prognostic index for the failure of in vitro fertilization.

This study had several limitations. First, the sample size included in this study was relatively small, which limited the detailed evaluation of the IVF fertilization rate. In addition, all subjects were from the same hospital, which restricted the generability of the study results. Studies with larger sample sizes were needed to further confirm the diagnostic value of the percentage of sperm with a small acrosome on fertilization failure.

## Conclusion

In conclusion, we found that the percentage of sperm with a small acrosome was positively correlated with the unexplained in vitro fertilization failure and negatively correlated with the in vitro fertilization rate. In addition, the percentage of sperm with a small acrosome could be used as a new prognostic factor for in vitro fertilization failure.

## Data Availability

All data generated or analyzed during this study are included in this. Further enquiries can be directed to the corresponding author.
